# Altered Pain Sensitivity in Elderly Women with Chronic Neck Pain

**DOI:** 10.1371/journal.pone.0128946

**Published:** 2015-06-03

**Authors:** Sureeporn Uthaikhup, Romchat Prasert, Aatit Paungmali, Kritsana Boontha

**Affiliations:** 1 Department of Physical Therapy, Faculty of Associated Medical Sciences, Chiang Mai University, Chiang Mai, Thailand; 2 Back, Neck and Other Joint Pain Research Group, Khon Kaen University, Khon Kaen, Thailand; West Virginia University School of Medicine, UNITED STATES

## Abstract

**Background:**

Age-related changes occur in both the peripheral and central nervous system, yet little is known about the influence of chronic pain on pain sensitivity in older persons. The aim of this study was to investigate pain sensitivity in elders with chronic neck pain compared to healthy elders.

**Methods:**

Thirty elderly women with chronic neck pain and 30 controls were recruited. Measures of pain sensitivity included pressure pain thresholds, heat/cold pain thresholds and suprathreshold heat pain responses. The pain measures were assessed over the cervical spine and at a remote site, the tibialis anterior muscle.

**Results:**

Elders with chronic neck pain had lower pressure pain threshold over the articular pillar of C5-C6 and decreased cold pain thresholds over the cervical spine and tibialis anterior muscle when compared with controls (*p* < 0.05). There were no between group differences in heat pain thresholds and suprathreshold heat pain responses (*p* > 0.05).

**Conclusion:**

The presence of pain hypersensitivity in elderly women with chronic neck pain appears to be dependent on types of painful stimuli. This may reflect changes in the peripheral and central nervous system with age.

## Introduction

Age-related change occurs in pain responses and pain processing. When healthy older persons are compared to young adults, increased pain thresholds are commonly reported [1, 2]. Several factors may account for changes in pain sensitivity with age, including a loss of nociceptors, a reduction in pain transmission input, impairments in peripheral and central nervous system functions and changes in the skin with age [1, 3, 4]. In addition, psychological distress and elders previous pain experiences may influence pain sensitivity [5].

While there is some knowledge of age effects on pain sensitivity, there is limited knowledge of the effects of chronic pain on measures of pain sensitivity in older persons. Two studies of produced conflicting results [6, 7]. One demonstrated no difference in pain sensitivity measures (pressure and cold pain thresholds) in elders with and without frequent intermittent headache [7], whereas a study of older persons with knee osteoarthritis found localized and generalized pain sensitivity (pressure and heat pain thresholds) [6]. Notably, suprathresholds have not been evaluated in elders with chronic pain. These may be a better measure, given the changes in the skin and loss of nociceptors with age. Thus further research into pain sensitivity in older persons with chronic pain is required. A better understanding of chronic pain mechanisms in the older population may lead to better management of this age group.

Neck pain is a common musculoskeletal problem in the elderly population and was chosen as a clinical model of chronic pain for this study. There is evidence of reductions in pressure and thermal (heat and cold) pain thresholds over the cervical spine in various populations of young to middle aged adults with neck pain [8–10]. Some studies have also demonstrated lower pain thresholds in areas outside the injured site [9, 10]. The heightened pain responses over both the site of pain/injury and areas remote to the injured site suggest alterations of peripheral and central nervous pain processing. However, data from these younger populations may not generalize to elders with chronic neck pain. The purpose of this study was to determine pain sensitivity using quantitative sensory testing (QST) of mechanical and thermal thresholds and thermal suprathresholds in female elders with chronic neck pain compared to controls without neck pain. Female elders only were investigated in this study as gender may influence pain thresholds [11]. As psychological factors are commonly associated with chronic pain and pain sensitivity [12], measures of depression and anxiety were included. We hypothesized that the presence of chronic pain would alter both threshold and suprathreshold measures of mechanical and thermal pain in elders.

## Materials and Methods

### Participants

Sixty female elderly participants (30 with neck pain and 30 healthy controls), aged between 65–75 years were recruited into the study from the physiotherapy outpatient clinics and advertising in the general community in a regional city in Thailand. Participants were eligible for the neck pain group if they had suffered from idiopathic neck pain for at least 3 months and scored equal to or greater than 10 (out of a possible 100) on the Neck Disability Index-Thai version (NDI-TH) scale [13]. Participants in the control group had no history of neck pain requiring treatment in the last 12 months. Potential participants were excluded if they had a history of neck trauma or injury, any musculoskeletal problems/disorders that would have an effect on measures and for which they had sought for medical treatment (e.g. fibromyalgia, rheumatoid arthritis), neurological abnormalities (e.g. numbness, pin and needles) or diseases (e.g. stroke, Alzheimer’s disease). All eligible participants were asked to refrain from taking analgesic/muscle relaxant medications on the day of testing.

Ethic clearance for the study was gained from the Human Ethical Review Board of the Faculty of Associated Medical Sciences, Chiang Mai University and all procedures were conducted in accordance with the declaration of Helsinki. Written informed consent was obtained from each patient before participation.

### Neck Disability Index-Thai version (NDI-TH)

The NDI-TH was used to measure self-reported levels of neck pain and disability. The NDI-TH consists of 10 items designed to assess pain intensity, personal care, lifting, reading, headache, concentration, work, driving, sleeping and recreation. The score for each item ranges from 0 to 5, with 5 denoting the lowest level of function. The total score was calculated as the sum of the scores obtained from each item. A higher score of the NDI-TH suggests greater disability associated with the neck pain. The NDI-TH was shown to be a valid and reliable tool for use in Thai populations with neck pain [13, 14].

### Visual Analogue Scale (VAS)

The VAS (0–10) was used to assess the intensity of neck pain when at its worst, on average and on the testing day.

### Thai Geriatric Depression Scale (TGDS)

The TGDS was used to measure self-rated depressive symptoms. The TGDS consists of 30 items of the original scale. A total score ≥ 13 indicates depressive symptoms. The validity of TGDS was shown to be excellent [15, 16].

### State Trait Anxiety Inventory-Thai version (STAI-TH)

The STAI-TH was used to measure the severity of the overall anxiety level. The STAI-TH is divided into two sections: the state and trait anxiety. Each section consists of 20 questions. Participants rated their anxiety on a 4-point Likert scale, where 1 = not at all and 4 = mostly. The range of total scores for each section is 20–80, with higher scores indicating a greater level of anxiety. The STAI-TH was translated to Thai from the original English version and the reliability was shown to be good [17].

### Pressure pain thresholds (PPTs)

Pressure pain thresholds were measured with an electronic digital algometer according to the methods described by Scott et al [18]. The algometer consists of a 1 cm diameter round rubber tip connected to a pressure transducer within the handle of the unit. Pressure was applied at a constant rate of 40 kPa/s. Participants were instructed to press a button when the pressure sensation under the probe was perceived as pain. The PPTs were tested over the articular pillars of C5-C6 and the tibialis anterior muscle (upper one-third of the muscle belly), a remote site, bilaterally three times and the mean values were used for analysis. A 30-second interval was allowed between measures.

### Thermal pain thresholds (TPTs)

Heat and cold pain thresholds were tested with TSA-II Neurosensory Analyzer according to the method described by Scott et al [18]. A Peltier thermode (30x30 mm) was applied directly over the skin. The baseline thermode temperature was set at 30°C with the rate of temperature change being 1°C/s. The maximum cut-out temperature of 50°C was set for heat pain threshold and of 0°C for cold pain threshold. Participants were instructed to press a patient-controlled switch when the heat or cold sensation under the probe was perceived as painful. If the warm or cold pain threshold was not reached before the cut-out temperature of 50°C or 0°C, the cut-out temperature of 50°C or 0°C were recorded for that trial. The heat and cold pain thresholds were measured over the mid-cervical region and the tibialis anterior muscle bilaterally three times and the mean values were used for analysis. A 30-second interval was allowed between measures.

### Suprathresholds

Suprathresholds are ratings of painful stimulation above threshold [19]. The suprathreshold was tested with TSA-II Neurosensory Analyzer. A Peltier thermode (30 x 30 mm) was applied directly to the skin. The baseline temperature for each pulse was set at 35°C and increased at a rate of 4°C/s. Three heat pulses (45°C, 47°C and 49°C) were used in random order and each pulse was kept constant for five seconds before returning to baseline. A 10-second pause was allowed between each pulse. Participants were instructed to rate each pulse using a numerical rate scale (NRS) ranging from 0 (no pain) to 100 (worst pain imaginable). Supra-threshold heat pain ratings were measured bilaterally over the mid-cervical region and the tibialis anterior muscle (upper one-third of the muscle belly). Measures were taken twice and the mean values were used for analysis.

### Procedure

Participants completed the questionnaires. The QST measures were tested in a quiet and temperature-controlled laboratory (24°C ± 1°C) in a standard order: PPTs, TPTs (heat and cold) and suprathreshold tests. Participants were positioned in prone position for measures over the cervical region and in the supine position for measures over the tibialis anterior muscle. A familiarization session was conducted using a site over the medial side of the forearm. The assessor was blinded to the participants’ neck pain or control status for all tests.

### Statistical analysis

All QST data were tested for normality using Kolmogorov-Smirnov test. Paired *t*-test was used to determine differences between sides for the PPTs and Wilcoxon Signed Ranks Test for the TPTs and suprathresholds. There were no differences between sides in the PPTs, TPTs and suprathresholds (all *p* > 0.05). The mean values of both sides for the QST were used for between-group comparisons. Univariate analyses were used to analyze differences between groups for the PPTs and TPTs and Mixed model ANOVA for the suprathresholds. The psychological features (TGDS and STAI-TH scores) and the presence of co-morbid musculoskeletal pain were included as covariates to adjust for differences in the QST. The significant level was set at p < 0.05. All statistical analyzes were conducted using SPSS for Windows (version 17.0).

## Results

### Participants

Participant demographics and neck pain characteristics are presented in Table 1. There were no significant differences between the groups in age and BMI (*p* > 0.05).

**Table 1:** Demographic data of participants.

	Neck pain	Controls
Age (yrs)	69.2 ± 3.7	70.6 ± 3.4
Body mass index (kg/m^2^)	24.2 ± 3.5	24.4 ± 3.7
History of neck pain (mo)	41.3 ± 76.5	-
NDI (0–100)	24.8 ± 12.2	-
VAS (0–10)		
on the testing day	4.2 ± 2.5	-
on average	4.4 ± 2.1	-
pain at its worst	6.7 ± 2.4	-
Co-morbid musculoskeletal pain (n)	24	19
TGDS (0–30)	5.0 ± 4.2	3.2 ± 3.3
STAI-state score (20–80)	35.5 ± 8.8	32.7 ± 7.1
STAI-trait score (20–80)	34.1 ± 8.0	32.7 ± 7.6

Values are presented as mean ± SD unless otherwise indicated

### Pressure pain thresholds (PPTs)

Elders with neck pain had significantly lower PPT over the articular pillar of C5-C6 compared to controls (*p* < 0.05, *η* ^*2*^ *p* = 0.25) (Table 2). No significant difference was found in PPT over the tibialis anterior muscle between the two groups (*p* > 0.05, *η* ^*2*^ *p* = 0.002).

**Table 2:** Pain thresholds and suprathresholds between elders with and without neck pain.

	Cervical spine		Tibialis anterior	
	Neck pain	Controls	Neck pain	Controls
Pain Thresholds				
PPTs (kPa)	287.0 ± 58.0	376.5 ± 81.4*	482.2 ± 129.0	488.3 ± 119.5
HPTs (°C)	47.3 ± 2.7	47.8 ± 1.1	47.9 ± 1.4	48.5 ± 0.8
CPTs (°C)	3.0 ± 3.5	0.5 ± 1.0*	1.5 ± 3.2	0.1 ± 0.3*
Suprathresholds (NRS 0–100)				
45°C	64.4 ± 22.7	66.6 ± 21.2	53.3 ± 20.1	49.6 ± 19.4
47°C	74.2 ± 19.8	74.6 ± 20.8	71.3 ± 17.3	68.0 ± 18.3
49°C	92.3 ± 16.5	89.3 ± 20.0	93.0 ± 14.5	89.8 ± 19.8

PPTs = pressure pain thresholds, HPTs = Heat pain thresholds, CPTs = Cold pain thresholds

Values are presented as mean ± SD, NRS = Numerical rating scale

* p < 0.05 compared between the neck pain and control groups

### Thermal pain thresholds (TPTs)

Elders with neck pain had significantly lower CPTs over the cervical spine and tibialis anterior muscle (*p* < 0.05, *η* ^*2*^ *p* = 0.20 and 0.09, respectively) when compared with elders without neck pain (Table 2). There were no significant between group differences in HPTs over the cervical spine and tibialis anterior muscle (*p* > 0.05, *η* ^*2*^ *p* = 0.008 and 0.05, respectively).

### Suprathresholds

There were no significant differences between groups for supra-threshold heat pain ratings over the cervical spine and tibialis anterior muscle (*p* > 0.05, *η* ^*2*^ *p* = 0.000 and 0.01, respectively) (Table 2). Interactions between group and suprathershold were not found (*p* > 0.05).

## Discussion

The results of this study partially support our hypothesis. Elders with chronic neck pain had decreased pain thresholds to pressure and cold stimuli but not to heat stimuli (both pain thresholds and suprathresholds). This may suggest that the presence of increased sensitivity in elders with chronic neck pain depends on types of painful stimuli.

The reduction in PPT and CPT over the cervical spine and CPT over the tibialis anterior muscle in our elders with chronic neck pain are in accordance with findings of previous studies conducted in younger populations with chronic neck pain [8, 9, 18]. It has been suggested that differences of between 17%-33% in PPT measurements over the cervical region are required to demonstrate a true clinical change in thresholds [20]. The mean PPT in our elders with chronic neck pain exhibited approximately a 24% decrease. Decreased PPT and CPT over the cervical spine (a local site) may indicate peripheral nociceptor sensitization, as a consequence of cervical arthopathy in this group [21]. Arthopathies may be associated with peripheral inflammation and an increase in pain substances (e.g. substance P, bradykinin, prostaglandins) [22]. Widespread hypersensitivity to cold stimuli could suggest the presence of central sensitization, which may be related to impaired central nervous system functions, in particular impaired descending inhibitory systems [23]. However, we found no evidence of lower PPTs at remote sites (tibialis anterior) which could be expected in the presence of a centrally sensitized state.

The study demonstrated no increased HPT in elders with chronic neck pain, which is comparable to previous studies of younger persons with neck pain [9, 18]. The difference in CPT but not HPT may reflect different mechanisms in the processing of painful stimuli. Although heat and cold pain stimuli are mediated by both Aδ fibers (myelinated fibers) and C-fibers (unmyelinated fibers), heat pain stimuli are predominantly mediated by C-fibers, which have slow conduction velocity and sensitivity [24].

There has been little research investigating suprathresholds responses to heat stimuli in elders with and without pain. Suprathreshold responses are thought to be associated with the presence of central sensitization [25, 26]. We found no differences in suprathreshold heat pain scores between elders with and without chronic neck pain. Taken together, the suprathreshold heat pain responses and the HPT results may suggest that central sensitization is probably not a feature of chronic neck pain in the elderly.

Pain thresholds may be confounded by age-related changes in the sensory system [1]. There are age-related changes in the peripheral and central nervous systems, including a reduction in the number and density of myelinated and unmyelinated fibers [27, 28], a smaller primary somatosensory cortex [29] and an impaired endogenous pain inhibitory system [3]. Numerous studies have demonstrated that older persons have increased pain thresholds compared to younger persons [4]. Accordingly, the mean values of pain thresholds to either mechanical (pressure) or thermal (heat and cold) in our elders (both with and without neck pain) were relatively higher than those obtained in younger persons [9]. For example, in a study by Javanshir et al [9], using similar instruments and methodologies, the mean PPT, CPT and HPT values at the cervical spine in younger subjects without neck pain were 205.3 ± 75.1 kPa, 10.4 ± 7.8°C and 44.2 ± 2.1°C, respectively. In our study, the mean PPT, CPT and HPT values at the cervical spine in elders without neck pain were 376.5 ± 81.4 kPa, 0.5 ± 1.0°C and 47.8 ± 1.1°C, respectively. The discrepancy between the results may be the consequence of age-related changes in either the peripheral or the central nervous system or both.

We found no differences in TGD scores between elders with and without neck pain. The mean TGDS score in elders with neck pain was marginally higher than that in elders without neck pain, but scores well below threshold values indicating depression (TGDS score > 13/30). In addition, neither depression nor anxiety influenced pain sensitivity which is consistent with previous findings in young/middle-age individuals with neck pain [18, 30] and elders with headache [7]. Previous studies have also found no influence of anxiety on mechanical and thermal pain thresholds and no correlations between pain thresholds and anxiety scores [18, 30]. There is evidence suggesting that older persons are more accepting of their pain and some may have increased emotional control [31, 32]. This may explain why psychological distress was not greater in our elders with neck pain compared to those without neck pain.

The results of this study suggest that elders with chronic neck pain have some evidence of increased pain sensitivity but interestingly, it depends on types of painful stimuli. Lower pressure and cold pain thresholds were found in elders with chronic neck pain but there were no changes in heat pain thresholds and suprathresholds. The reason for this is unclear but it may be associated with age-related changes in the peripheral and central nervous system functions. Future research is required to investigate these differences towards a better understanding of pain processing mechanisms in older persons with chronic pain. Some limitations should be addressed in the study. The effect sizes of the negative results were small. Nevertheless a larger sample size is required to confirm the findings of this study. Musculoskeletal pain is common in older persons [33]. Thus it was difficult to recruit elderly participants who have chronic neck pain alone. The experience of pain in those elders may also influence the expectation and pain outcomes. Additionally, chronic use of opioids may influence pain sensitivity.

## Supporting Information

S1 Dataset The data for pain thresholds and suprathresholds for elders with and without neck pain (XLSX)

## References

[1] Gibson SJ, Farrell M. A review of age differences in the neurophysiology of nociception and the perceptual experience of pain. Clin J Pain. 2004;20:227–239. 15218407 10.1097/00002508-200407000-00004

[2] Lautenbacher S. Experimental approaches in the study of pain in the elderly. Pain Med. 2012;13 Suppl 2:S44–50. 10.1111/j.1526-4637.2012.01326.x 22497747

[3] Edwards RR, Fillingim RB, Ness TJ. Age-related differences in endogenous pain modulation: a comparison of diffuse noxious inhibitory controls in healthy older and younger adults. Pain. 2003;101:155–165. 12507710 10.1016/s0304-3959(02)00324-x

[4] Gibson SJ, Helme RD. Age-related differences in pain perception and report. Clin Geriatr Med. 2001;17:433–456. 11459714 10.1016/s0749-0690(05)70079-3

[5] Herr KA, Garand L. Assessment and measurement of pain in older adults. Clin Geriatr Med. 2001;17:457–478. 11459715 10.1016/s0749-0690(05)70080-x PMC3097898

[6] Lee YC, Lu B, Bathon JM, Haythornthwaite JA, Smith MT, Page GG, et al Pain sensitivity and pain reactivity in osteoarthritis. Arthritis Care Res. 2011;63:320–327. 10.1002/acr.20373 20957660 PMC3030930

[7] Uthaikhup S, Sterling M, Jull G. Widespread sensory hypersensitivity is not a feature chronic headache in elders. Clin J Pain. 2009;25:699–704. 10.1097/AJP.0b013e3181a38f88 19920720

[8] Chien A, Sterling M. Sensory hypoaethesia is a feature of chronic whiplash but not chronic idiopathic neck pain. Man Ther. 2010;15:48–53. 10.1016/j.math.2009.05.012 19632884

[9] Javanshir K, Ortega-Santiago R, Mohseni-Bandpei MA, Miangolarra-Page JC, Fernández-de-las-Peñas C. Exploration of somatosensory impairments in subjects with mechanical idiopathic neck pain: a preliminary study. J Manipulative Physiol Ther. 2010;33:493–499. 10.1016/j.jmpt.2010.08.022 20937427

[10] Johnston V, Jimmieson NL, Jull G, Souvlis T. Quantitative sensory measures distinguish office workers with varying levels of neck pain and disability. Pain. 2008;137:257–265. 17964075 10.1016/j.pain.2007.08.037

[11] Bartley EJ, Fillingim RB. Sex differences in pain: a brief review of clinical and experimental findings. Br J Anaesth. 2013;111:52–58. 10.1093/bja/aet127 23794645 PMC3690315

[12] Wallin M, Liedberg G, Borsbo B, Gerdle B. Thermal detection and pain thresholds but not pressure pain thresholds are correlated with psychological factors in women with chronic whiplash-associated pain. Clin J Pain. 2012;28:211–221. 10.1097/AJP.0b013e318226c3fd 21750459

[13] Uthaikhup S, Paungmali A, Pirunsan U. Validation of Thai versions of the Neck Disability Index and Neck pain and Disability Scale in patients with neck pain. Spine. 2011;36:1415–1421. 10.1097/BRS.0b013e31820e68ac 21228749

[14] Luksanapruksa P, Wathana-apisit T, Wanasinthop S, Sanpakit S, Chavasiri C. Reliability and validity study of a Thai version of the Neck Disability Index in patients with neck pain. J Med Assoc Thai. 2012;95:681–688. 22994028

[15] Committee TtBF. Thai Geriatric Depression Scale-TGDS. Siriraj Hosp Gaz. 1994;46:1–9.

[16] Thongtang O, Sukhatunga K, Ngamthipwatthana T, Chulakadabba S, Vuthiganond S, Pooviboonsuk P, et al Prevalence and incidence of depression in the Thai elderly. J Med Assoc Thai. 2002;85:540–544. 12188382

[17] Kotchabhakdi N, Vorakitphokatorn S, Nitsaisook M. The State-Trait Anxiety Inventory-Thai. National Institute for Child and Family Development, Mahidol University; 1983.

[18] Scott D, Jull G, Sterling M. Widespread sensory hypersensitivity is a feature of chronic whiplash-associated disorder but not chronic idiopathic neck pain. Clin J Pain. 2005;21:175–181. 15722811 10.1097/00002508-200503000-00009

[19] Robinson ME, Bialosky JE, Bishop MD, Price DD, George SZ. Supra-threshold scaling, temporal summation, and after-sensation: relationships to each other and anxiety/fear. J Pain Res. 2010;3:25–32. 21197307 10.2147/jpr.s9462 PMC3004634

[20] Prushansky T, Dvir Z, Defrin-Assa R. Reproducibility indices applied to cervical pressure pain threshold measurements in healthy subjects. Clin J Pain. 2004;20:341–347. 15322441 10.1097/00002508-200409000-00009

[21] Cavanaugh JM, Lu Y, Chen C, Kallakuri S. Pain generation in lumbar and cervical facet joints. J Bone Joint Surg. 2006;88:63–67. 16595446 10.2106/JBJS.E.01411

[22] Curatolo M, Arendt-Nielsen L, Petersen-Felix S. Central hypersensitivity in chronic pain: mechanisms and clinical implications. Phys Med Rehabil Clin N Am. 2006;17:287–302. 16616268 10.1016/j.pmr.2005.12.010

[23] Graven-Nielsen T, Babenko V, Svensson P, Arendt-Nielsen L. Experimentally induced muscle pain induces hypoalgesiain heterotopic deep tissues, but not in homotopic deep tissues. Brain Res. 1998;787:203–210. 9518613 10.1016/s0006-8993(97)01480-7

[24] Hansson P, Backonja M, Bouhassira D. Usefulness and limitations of quantitative sensory testing: Clinical and research application in neuropathic pain states. Pain. 2007;129:256–259. 17451879 10.1016/j.pain.2007.03.030

[25] Ashina S, Bendtsen L, Ashina M, Magerl W, Jensen R. Generalized hyperalgesia in patients with chronic tension-type headache. Cephalalgia. 2006;26:940–948. 16886930 10.1111/j.1468-2982.2006.01150.x

[26] Valencia C, Fillingim RB, Gerorge SZ. Suprathreshold heat pain response is associated with clinical pain intensity for patients with shoulder pain. J Pain. 2011;12:133–140. 10.1016/j.jpain.2010.06.002 20692209 PMC3697912

[27] Jacobs JM, Love S. Qualitative and quantitative morphology of human sural nerve at different ages. Brain. 1985;108:897–924. 4075078 10.1093/brain/108.4.897

[28] Kanda T, Tsukagoshi H, Oda M, Miyamoto K, Tanabe H. Morphological changes in unmyelinated nerve fibers in the sural nerve with age. Brain. 1991;114:585–599. 2004257 10.1093/brain/114.1.585

[29] Quiton RL, Roys SR, Zhuo J, Keaser ML, Gullapalli RP, Greenspan JD. Age-related changes in nociceptive processing in the human brain. Ann NY Acad Sci. 2007;1097:175–178. 17413021 10.1196/annals.1379.024

[30] Touche RL, Fernández-de-las-Peñas C, Fernández-Carnero J, Díaz-Parreñ S, Paris-Alemany A, Arendt-Nielsenz L. Bilateral mechanical-pain sensitivity over the trigeminal region in patients with chronic mechanical neck pain. J Pain. 2010;11:256–263. 10.1016/j.jpain.2009.07.003 19945351

[31] Sofaer B, Moore AP, Holloway I, Lamberty JM, Thorp TAS, O'Dwyer J. Chronic pain as perceived by older people: a qualitative study. Age Ageing. 2005;34:462–466. 16043446 10.1093/ageing/afi139

[32] Jorm AF. Does old age reduce the risk of anxiety and depression? a review of epidemiological studies across the adult life span. Psychol Med. 2000;30:11–22. 10722172 10.1017/s0033291799001452

[33] Brochet B, Michel P, Barberger-Gateau P, Dartigues JF. Population-based study of pain in elderly people: a descriptive survey. Age Ageing. 1998;27:279–284.

